# Thymic Medulla Epithelial Cells Acquire Specific Markers by Post-Mitotic Maturation

**DOI:** 10.1155/1996/61035

**Published:** 1996

**Authors:** Claude Penit, Bruno Lucas, Florence Vasseur, Theresa Rieker, Richard L. Boyd

**Affiliations:** 1 INSERM U. 345 Institut Necker 156 rue de Vaugirard Paris Cedex 15 75730 France; 2 Institute for General and Experimental Pathology University of Innsbruck Innsbruck A-6020 Austria; 3 Department of Pathology and Immunology Monash Medical School Commercial Road Prahran Melbourne Victoria Australia

**Keywords:** Thymus epithelium, thymocytes, cell proliferation, bone-marrow transfer, RAG-2-/– mice

## Abstract

The development of thymocyte subsets and of the thymic epithelium in SCID and RAG-2^-^/– mice was monitored after normal bone-marrow-cell transfer. The kinetics of thymic reconstitution and their relationships with cell proliferation were investigated by using bromodeoxyuridine to detect DNA-synthesizing cells among lymphoid cells by 3-color flow cytometry, and in epithelial compartments by staining frozen sections. Thymocytes started to express CD8 and CD4 10 days after transfer, simultaneously with extensive proliferation. The first mature CD4^+^ single-positive cells were generated, from resting CD4^+^CD8^+^ cells after day 15. During this day 10–15 period, many epithelial cells positive for cortexspecific or panepithelial markers were labeled with BrdUrd after pulse-injection. Organized medullary epithelium also developed after day,15, that is, synchronously with the appearance of mature thymocytes, but medullary cells were never found BrdUrd^+^. These results suggest that, in these models, the reconstitution of the thymic epithelial network proceeds through expansion of preexisting cortical or undifferentiated cells and by later maturation (acquisition of specific markers) of medullary cells. This last process is dependent of the presence of mature thymocytes.


*Developmental Immunology*, 1996, Vol 5, pp. 25–36
Reprints available directly from the publisher
Photocopying permitted by license only

© 1996 OPA (Overseas Publishers Association)
Amsterdam B.V. Published in The Netherlands
by Harwood Academic Publishers GmbH
Printed in Singapore

### Thymic Medulla Epithelial Cells Acquire Specific Markers by Post-Mitotic Maturation

CLAUDE PENIT,*† BRUNO LUCAS,† FLORENCE VASSEUR,† THERESA RIEKER,‡ and RICHARD L. BOYD§

†*INSERM U. 345, Institut Necker, 156 rue de Vaugirard, 75730 Paris Cedex 15, France*
‡*Institute for General and Experimental Pathology, University of Innsbruck, A-6020 Innsbruck, Austria*
§*Department of Pathology and Immunology, Monash Medical School, Commercial Road, Prahran, Melbourne, Victoria, Australia*

The development of thymocyte subsets and of the thymic epithelium in SCID and RAG-$2^{-}$/– mice was monitored after normal bone-marrow-cell transfer. The kinetics of thymic reconstitution and their relationships with cell proliferation were investigated by using bromodeoxyuridine to detect DNA-synthesizing cells among lymphoid cells by 3-color flow cytometry, and in epithelial compartments by staining frozen sections. Thymocytes started to express CD8 and CD4 10 days after transfer, simultaneously with extensive proliferation. The first mature $CD4^{+}$ single-positive cells were generated, from resting $CD4^{+}CD8^{+}$ cells after day 15. During this day 10–15 period, many epithelial cells positive for cortex-specific or panepithelial markers were labeled with BrdUrd after pulse-injection. Organized medullary epithelium also developed after day 15, that is, synchronously with the appearance of mature thymocytes, but medullary cells were never found $BrdUrd^{+}$. These results suggest that, in these models, the reconstitution of the thymic epithelial network proceeds through expansion of preexisting cortical or undifferentiated cells and by later maturation (acquisition of specific markers) of medullary cells. This last process is dependent of the presence of mature thymocytes.

KEYWORDS: Thymus epithelium, thymocytes, cell proliferation, bone-marrow transfer, RAG-$2^{-}$/– mice.

### INTRODUCTION

The role of thymic stromal cells in thymocyte differentiation and selection is well documented (Van Ewijk, 1991; Jenkinson et al., 1992; Ritter and Boyd, 1993), although the precise mechanisms of lymphostromal interactions are far from being clearly understood. The inverse relationship, that is, the role of lymphoid cells in the organization of the thymic stroma, has been demonstrated by the observation of the thymus of SCID (Bosma and Carroll, 1991) and RAG-$2^{-}$/– (Shinkai et al., 1992) mice, which bear natural or artificially induced mutations of the T-cell receptor (TCR) gene-rearrangement system, and are thus incapable of T-cell production. In these mice, thymocyte development is arrested at the $CD44^{-}$ $CD25^{+}$ stage of triple-negative ($TCR^{-}CD4^{-}CD8^{-}$) precursors (Godfrey et al., 1993; Penit et al., 1995), and the thymic stroma lacks structural organization: Cortical or panepithelial markers can be detected by specific monoclonal antibodies, but medullary epithelial cells are very scarce or absent (Shores et al., 1991; Holländer et al., 1995). The thymic architecture can be restored by reconstitution of SCID or RAG-$2^{-}$/– mice with normal bone marrow (Shores et al., 1991) but also after injection of mature lymphocytes (Surh et al., 1992; Hilbert et al., 1993), suggesting that the presence of mature thymocytes is necessary and sufficient for medullary stroma development. Medullary epithelial growth in these systems can be due to either proliferation of rare and dispersed cells present in the mutant thymus or to the acquisition of medulla-specific markers, without proliferation, by epithelial cells in contact with mature thymocytes. To resolve this question, we employed the BrdUrd method that we have established to study the kinetics of thymocyte differentiation (Pénit and Vasseur, 1988; Lucas et al., 1993), maturation (Lucas et al., 1993), and selection (Lucas et al., 1994). We simultaneously determined the kinetics of thymocyte subset reconstitution, and the phenotype of cycling thymocytes in RAG-$2^{-}$/– mice after i.v. injection of bone-marrow cells, by analyzing BrdUrd

*Corresponding author.

and surface staining of thymocytes at different time points after BM injection, by three-color cytometry. Sections from the same thymuses were stained with a series of epithelial or lymphoid markers and BrdUrd. The results show that extensive proliferation of cortical and/or nondifferentiated (putative precursors) epithelial cells is observed in parallel to lymphoid expansion, but that subsequent medullary growth is independent of cell proliferation.

### RESULTS

### Thymocyte Development in BM-Reconstituted RAG-2⁻/– Mice

Mice deficient for the RAG-2 gene are unable to rearrange and express TCR genes. Consequently, the thymuses of these mice contain only precursor cell subsets, that is, $CD3^-CD4^-CD8^-$ cells, that stop development at the $CD44^-CD25^+HSA^+$ stage (Fig. 1, top, and Mombaerts et al., 1992; Shinkai et al., 1992). Normal bone-marrow cells injected i.v. into low-dose irradiated $RAG\text{-}2^-/-$ mice develop normally and generate all normal thymocyte and peripheral T-cell subsets. Three transfer experiments were performed, and the kinetics of thymus reconstitution obtained are shown in Figs 1 and 2. Kinetics of precursor thymocytes were studied using CD44/CD25/BrdUrd staining (Fig. 1). The loss of CD25 by $CD3^-CD4^-CD8^-$ (triple-négative, TN) thymocytes is dependent on productive rearrangement of TCRβ-chain genes. This phenotypic change is accompanied by an intense cell proliferation and immediately precedes CD8, and then CD4 expression (Lucas et al., 1993; Pénit et al., 1995). Both CD25 loss and DNA synthesis in $CD44^-CD25^{lo/-}$subsets were observed on day 11 after $BM^-$ cell transfer (Fig. 1), simultaneously with the first appearance of $CD4^+8^+$ cells (Fig. 2). Transitions in the TN subset were then masked by the accumulation of $CD44^-CD25^-$ cells, including all $CD4/8^+$ thymocytes.

CD4/CD8 subset reconstitution was monitored from day 8 to day 21 post-BM transfer using both CD4/CD8/TCRβ and CD4/CD8/BrdU staining. Intermediate cells in the DN–DP transition comprised $CD4^-CD8^{lo}$, $CD4^-CD8^+$ and $Cd4^{lo}CD8^+$ cells that express no or very low levels of αβ-TCR. They were gated in a single region (Fig. 2) and will be referred as pre-DP subsets. They appeared on days 11–13, and reached a normal percentage (Fig. 2) and absolute number (not shown) by day 19. $CD4^+CD8^+$ thymocytes showed similar reconstitution kinetics. Finally, $CD4^+CD8^{lo}$ and $CD4^+CD8^-$ cells were detectable after day 15, and displayed their normal representation on day 21. The maturation status of single-positive and intermediate subsets was assessed by CD4/CD8/TCRβ staining (Fig. 3). As soon as their first appearance (day 15), $CD4^+CD8^{lo}$ and $CD4^+CD8^-$ cells were $TCR^{hi}$ in majority (60% on day 15, 80% on day 19), but still expressed a high HSA density on day 19 (data not shown). By contrast, mature $TCR^{hi}CD4^-CD8^+$ cells were completely absent on day 15, were first detected in significant percentages on day 19, and were still underrepresented on day 21. These reconstitution kinetics are in accord with the normal sequence of thymocyte-subset generation, which shows a 2-day delay between $CD4^+8^-$ and $CD4^-8^+$ cell generation (Lucas et al., 1993), and are very close to those observed in normal BM-radiation chimeras (Pénit and Ezine, 1989). Indeed, in those animals, thymocyte-subset generation proceeds through proliferation of pre-DP precursors, cell proliferation stops at the DP stage, and the subsequent generation of mature SP cells is essentially proliferation-independent. This process also occurred in reconstituted $RAG\text{-}2^-/-$ mice, as shown by measuring the DNA-synthesis rate after BrdUrd pulse labeling (Fig. 4). Thymocyte DNA-synthesis frequency peaked on day 13 and returned to normal values in all subsets by day 19. The proliferation rate of $CD4^+CD8^{lo}$ and $CD4^+CD8^-$ cells was always found to be low (less than 2%). The very high proliferation rate of pre-DP intermediates (70–80% on days 13–15) only concerned cells with null or very low TCR expression, in chimeras and normal mice, as recently confirmed by four-color labeling (not shown).

The distribution of cycling thymocytes was also studied on thymus frozen sections. However, under our labeling conditions, only one thymocyte marker can be detected, besides BrdUrd, on sections. These staining experiments were also done with reconstituted SCID mice. We have previously shown (Pénit, 1986, 1988) that normal DNA-synthesizing thymocytes are almost exclusively located in the cortex, with the highest frequency in the subcapsular region (SC). IL2-Rα-positive cycling cells are located in the SC cortex. Some rare $IL2\text{-}R^+$ cells are also scattered in the inner cortex, but most of them are not BrdUrd-labeled.

In the present study, the same double-color stainings were performed on bone-marrow (BM)-injected SCID or $RAG\text{-}2^-/-$ mouse thymuses. The results were essentially the same in both recipient

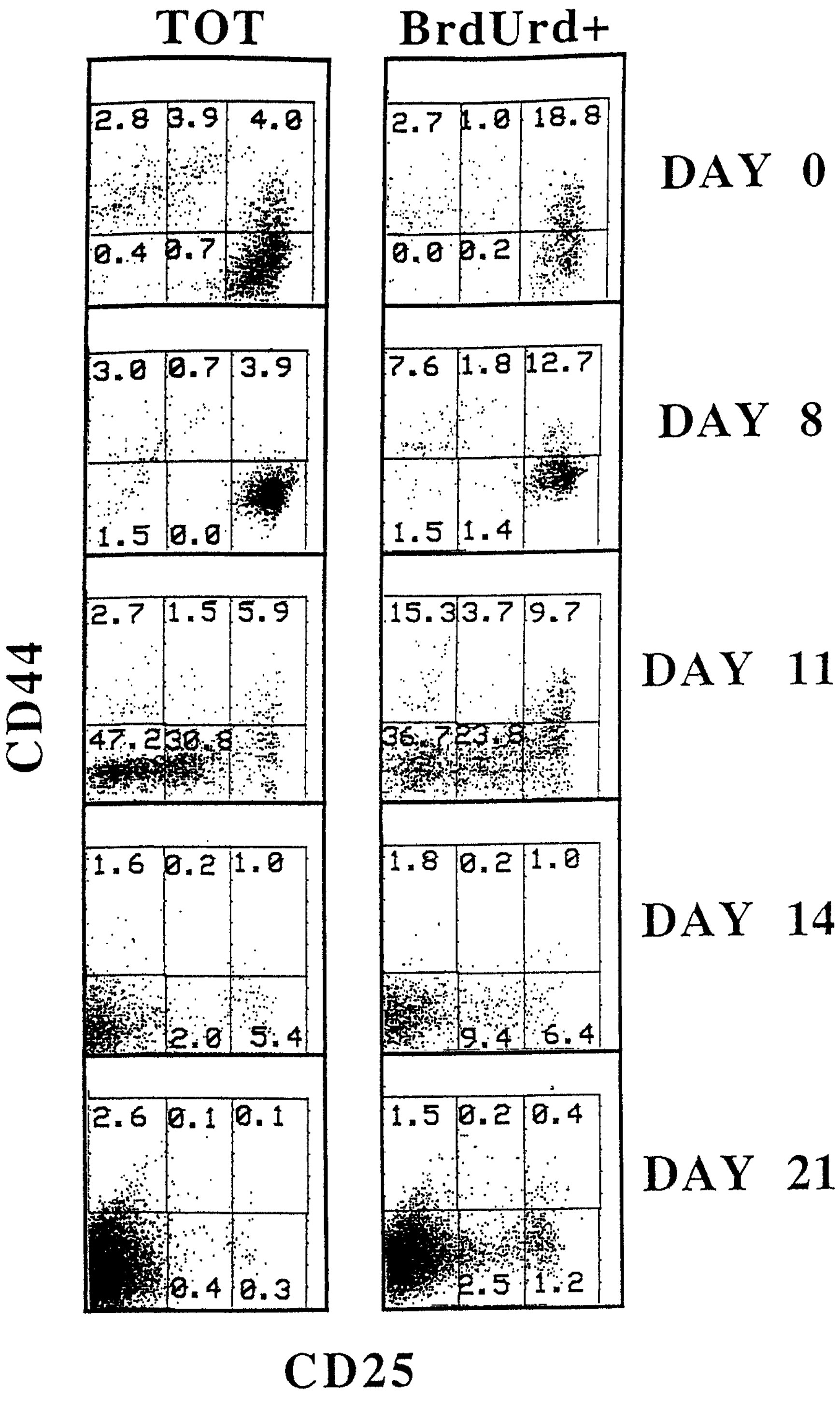

FIGURE 1. Development of thymocytes in BM-transferred RAG-2-/- mice: CD44/CD25 subsets. Thymuses of untreated (day 0) and BM-transferred (days 8, 11, 14, and 21) mice were taken 1 h after BrdUrd pulse and thymocyte suspensions were stained with anti-CD44-PE and biotinylated anti-CD25 revealed with Tricolor-streptavidin. BrdUrd was then detected with the 76-7 antibody and FITC-conjugated anti-mouse IgG1. Dot plots and numbers indicated in defined regions represent the subset distribution in total (left) and DNA-synthesizing (right) thymocytes. Downregulation of CD25 is clearly apparent on day 11, and the corresponding $CD25^{lo}$ and $CD25^{-}$ subsets are enriched in cycling cells.

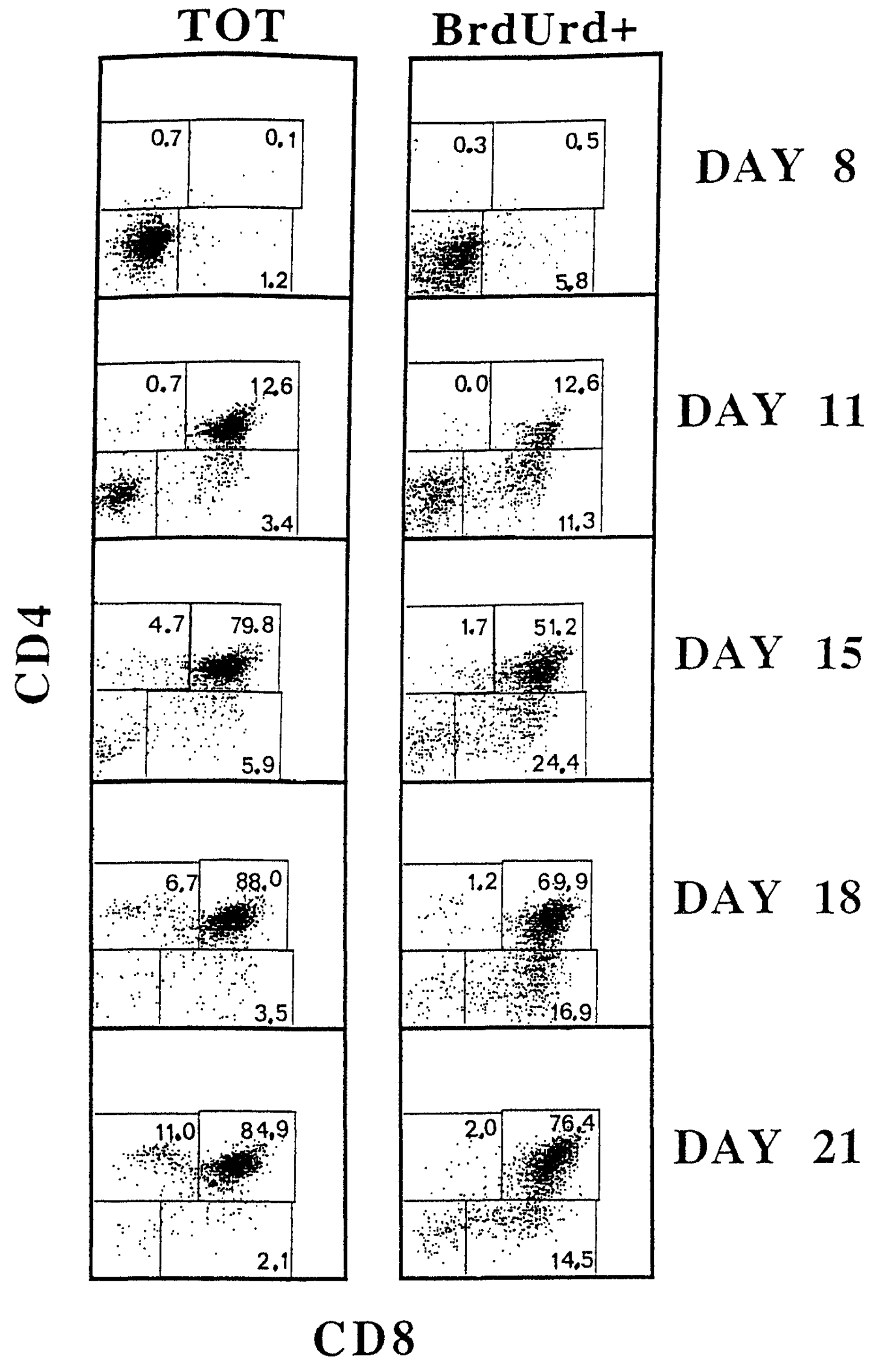

FIGURE 2. Development of thymocytes in BM-transferred RAG-2-/- mice: CD4/CD8 subsets. Same protocol as in Fig. 1. Surface staining was done with PE-anti CD4 and biotinylated anti-CD8 plus TC-streptavidin. CD8 and CD4 expression starts to be detectable on day 11, and corresponding intermediate subsets are enriched in cycling cells at this time and thereafter.

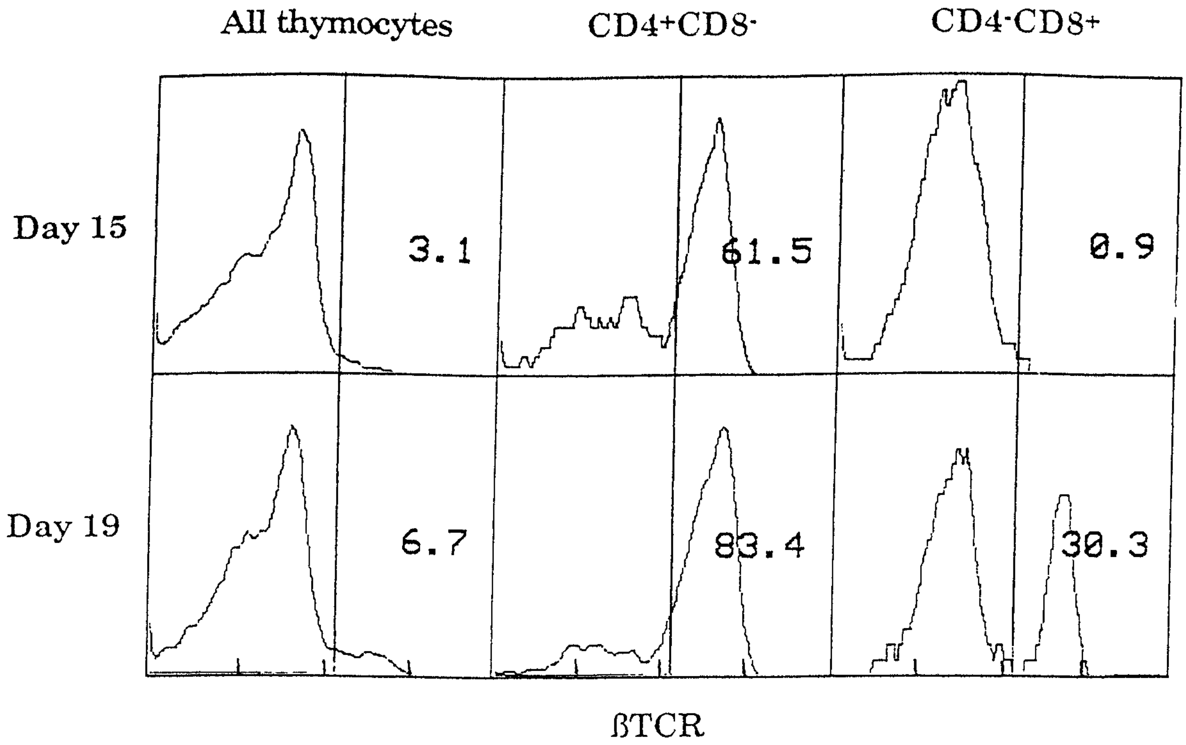

FIGURE 3. TCRβ chain expression in thymocytes from BM-reconstituted RAG-2⁻/– mice. Thymocytes harvested on day 15 and day 19 after BM-cell transfer were stained with anti-CD4-PE, anti-CD8-FITC, and biotinylated H57-597 anti-βTCR antibody revealed with Tricolor-streptavidin. The βTCR histogram obtained for unseparated (left), gated $CD4^{+}8^{-}$ (middle), and $CD4^{-}8^{+}$ (right) thymocytes are shown with the percentages of $TCR^{hi}$ cells.

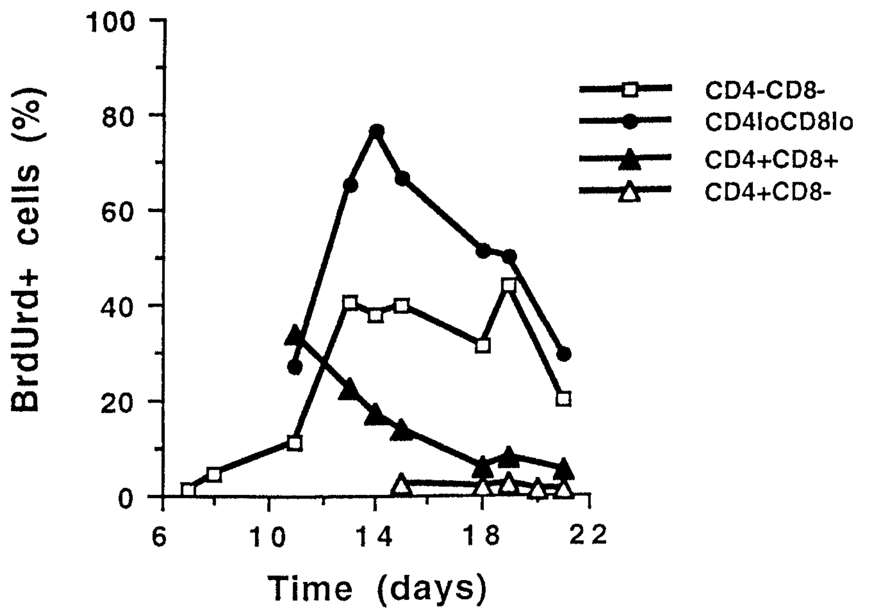

FIGURE 4. DNA-synthesis rate of thymocyte subsets in BM-transferred RAG-2⁻/– mice. Percentages of $BrdUrd^{+}$ cells were measured in CD4/CD8 subsets between day 7 and day 21 after transfer. Cells expressing CD8 and CD4 were first detected on day 11. DNA-synthesizing cell frequency in the total thymus was maximum on day 13.

systems and were in accord with the cytometry data. They are only briefly described here. On day 7 after BM injection, cycling cells were detected as large, irregularly shaped nuclei scattered inside the gland, without any preferential location. Interestingly, they were absent in the SC region, which then appears as the exclusive site of coreceptor induction. The sections were essentially negative for CD8 expression, and the very rare $CD8^+$ cells always were $BrdUrd^-$. Many IL-$2R\alpha^+$ cells were also found on day 8, some of which were $BrdUrd^+$, and most $BrdUrd^-$, but, anyway, the majority of $BrdUrd^+$ cells were IL-$2R\alpha^+$.

On day 10, thymus sections were still not stained by CD8 antibodies, and the number of $BrdUrd^+$ cells strongly increased, simultaneously with IL-$2R\alpha^+$ cells (not shown).

On day 15, cycling cells became very numerous and were distributed all over the section. CD8 expression was now extensive, and many $CD8^+$ cells were cycling. IL-$2R\alpha^+$ cell frequency had already dropped and positive cells were located mainly in $CD8^-$ regions.

### Thymic Epithelium Proliferation

In normal mouse thymus, cycling cortical thymocytes are surrounded by epithelial-cell processes detected by MTS 5 antibody. $BrdUrd^+$ nuclei are small, and always clearly separated from MTS $5^+$ epithelial cells. If $BrdUrd^+$ epithelial cells exist, they appear very infrequently. Similar pictures were obtained with MTS 39 (pan-epithelium) and MTS 44 (cortical epithelium-specific) antibodies. MTS 10 only stains medullary epithelium, and $BrdUrd^+$ cells were absent in MTS $10^+$ regions (data not shown).

On day 8, post-BM injection, SCID thymuses showed strong MTS 5 (Fig. 5a), MTS 39, and MTS 44 staining (not shown). Negative zones were also found, however. Among the relatively rare $BrdUrd^+$ cells found, some, but not all, were labeled with epithelial markers. On days 11 (Fig. 5b, MTS 44), the thymus was still hyperepithelial, and many cycling MTS $5^+$ or MTS $44^+$, but also MTS $39^+$ cells were observable. During the day 8–15 period, MTS $10^+$ cells were very scarce, and appeared as isolated scattered cells; they were always $BrdUrd^-$ (Figs 5d and 5e). On day 19 (Fig. 5f), small but typical MTS $10^+$ medullary zones were found, which contained only very few $BrdUrd^+$ nuclei. At this time, association of $BrdUrd^+$ nuclei with cortical epithelial cells resembled the normal situation (Fig. 5c) with absence of contact of labeled nuclei with epithelial MTS $5^+$ processes. MTS 44 staining showed negative areas, corresponding to MTS $10^+$ cells (data not shown).

Hence, the rapid growth of the thymic medulla was observed without significant proliferation of medullary-type epithelial cells.

The relationships between the nuclear BrdUrd green staining and the cytoplasmic epithelial red staining were more precisely analyzed using confocal microscopy. The analysis of a representative field of a section made on day 11 post-BM injection is shown in Fig. 6a. In the large photograph (left), three large labeled nuclei are observed, irregularly shaped and containing yellow patches corresponding to the superposition of red and green fluorescences. The small pictures correspond to 4 successive plans of increasing depth, and we can see that when visible, all three labeled nuclei retain the same aspect: close contact with red staining, with yellow patches. Other green nuclei of smaller size are also observed, which show only partial contacts with cytoplasmic epithelial staining, without yellow patches, and are likely to correspond to cycling thymocytes.

On day 19 (Fig. 6b), many labeled thymocyte nuclei were observed, uniformly green, with only occasional contacts with the epithelial MTS 5 staining. No cycling epithelial cells could be detected. Confocal microscope analysis of MTS 10/BrdUrd staining was done on day-17 sections (Fig. 6c), that is, at the same time as maximum medullary growth. The rare $BrdUrd^+$ nuclei found in MTS $10^+$ foci did not show any association with the cytoplasmic staining, suggesting that, like in normal thymus, medullary epithelial cells are resting during formation of the medulla.

### DISCUSSION

Using the BrdUrd labeling method, we determined the kinetics of thymocyte-subset reconstitution in BM transferred RAG-$2^-$/– mice. The release of the developmental block at the $CD44^-CD25^{hi}$ stage of TN precursors was observed on days 10–11, as shown by the successive appearance of $CD44^-CD25^{lo}$ and then $CD44^-CD25^-CD4^-CD8^{lo/+}$ cells. IL-$2R\alpha$ expression then dropped sharply, in parallel with the emergence of the $CD4^+8^+$ subset and the decrease of total mitotic activity. $CD4^-8^+$ cells showed a high proliferation rate, and were $TCR^{-/lo}$ until at least day 15. The first $CD4^+8^-$ cells emerged on day 15 and were also gathered in the subcapsular cortex, but positive cells scattered in the inner cortex (and often resting) were present at all late time points, as in the normal thymus.

Medullary foci, defined by MTS 10 expression developed in parallel with the appearance of $CD4^+8^-$ cells, that is, after day 15. In the absence of cell proliferation, the medullary growth could be due either to gathering of already present and dispersed medullary cells or to maturation of cortexlike or undifferentiated cells acquiring medulla-specific markers such as MTS 10. Simple reorganization and concentration appear unlikely because the rare MTS $10^+$ cells observed at early time points could not constitute the large medullary zones seen at days 19–21. The most probable mechanism, therefore, is the maturation of epithelial cells that are already present, expressing panepithelial, cortical, or unknown markers into medulla-specific cells, under the influence of recently matured thymocytes. This hypothesis is also supported by the occurrence of rare MTS 10–MTS 44 double-positive cells in the medulla (Boyd et al., unpublished data). Hence, the development of the thymic medulla is primarily due to maturation of epithelial cells that have previously expanded. These results are summarized in Fig. 7. This process appears to be dependent on the presence of mature cells, recently produced *in situ* in the present study or artificially transferred like in the work of Surh et al. (1992). The mechanism by which mature thymocytes induce maturation of the medullary epithelium remains unknown, but the present data show that the putative T-cell factors involved in this process are not simply mitogenic for preexisting epithelial cells, but are true maturation factors.

### EXPERIMENTAL PROCEDURE

### Mice

C57BL/6 mice were obtained from Iffa Credo (L'Arbresle, France), and used as controls and source of bone-marrow cells. CB17 scid/scid mice were bred

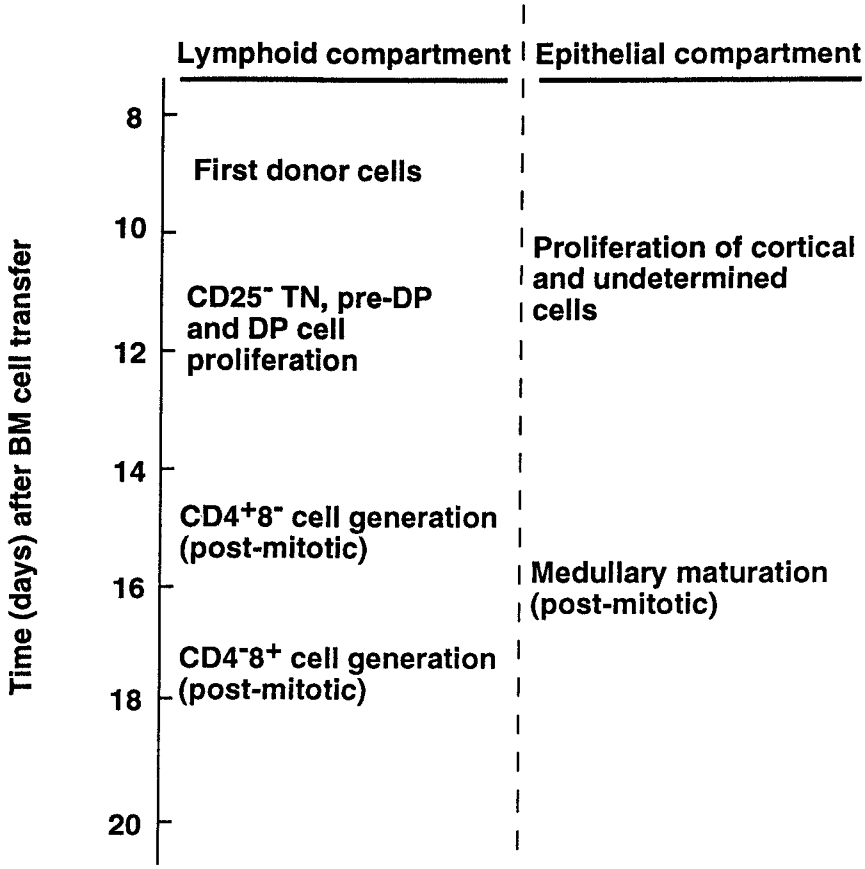

FIGURE 7. Development of lymphoid and epithelial cells in BM-reconstituted RAG-$2^-$/– mouse thymus.

in University of Innsbruck facilities. One hundred twenty-nine RAG-2⁻/− mice (Shinkai et al., 1992), obtained by courtesy of F.W. Alt, were bred at CDTA-CNRS (Orléans, France).

### Bone-Marrow Transfer

The work was started with SCID mice that were whole-body irradiated (3 Gy) and $10^7$ bone-marrow cells were injected i.v. 3 h later. To avoid any bias due to the leakiness of the scid mutation, we also used RAG-2⁻/− mice. RAG-2⁻/− mice were irradiated (3 Gy) and received $2 \times 10^7$ bone-marrow cells in a first series and $5 \times 10^6$ in a second one. In all cases, BM cells were thoroughly depleted of T cells with antibodies against CD3, CD4, and CD8 and magnetic beads.

Depending on the donor-cell number and on the recipient mouse strain (SCID or RAG-2⁻/−), small quantitative differences in thymic cellularity were observed, but thymic reconstitution was qualitatively and kinetically the same.

### BrdUrd Labeling and Thymus Section

Seven to 21 days after BM transfer, SCID and RAG-2⁻/− mice received two intraperitoneal injections of BrdUrd (1 mg each in 100 µl of PBS) at a 4-hr interval. One hour after the second injection, SCID thymuses were dissected, immediately frozen in liquid nitrogen, and stored at −80°C until use.

Frozen thymuses were embedded in Tissue Tek (Miles, Elkhart, Indiana) and Cryostat (Leica) cut sections (4 µm) were immediately fixed in 100% acetone and stored at −20°C.

Normal C57B1/6 mice were submitted to the same protocol and used as controls.

For each BM reconstituted RAG-2⁻/− mouse, one thymus lobe was frozen for immunochemistry and the other prepared as a cell suspension for flow cytometry.

### Immunofluorescence on Frozen Sections

Fixed sections were stained with anti-CD8 (clone 53–67), anti-Thy1.1 + 1.2, anti-Thy1.1 (19XE5), anti-IL2-Rα (PC61), MTS 5, MTS 10, MTS 12, MTS 39, MTS 44, or anti-Mac-1 (M1-70) revealed with biotinylated goat anti-rat Ig followed by Texas Red-streptavidin (Caltag).

Stained sections were fixed again in 70% ethanol and treated for BrdUrd detection, as described (Pénit, 1988). Briefly, semidried sections were incubated in 4N HCl, neutralized with Borax, and washed in PBS. BrdUrd was detected with the 76/7 moAb (a gift of T. Ternynck, Institut Pasteur, Paris) revealed with FITC-conjugated goat anti-mouse IgG1 (Southern Biotechnologies). All incubations with antibodies were done for 30 min at 4°C except for anti-BrdUrd (at 20°C). Sections were observed under an Orthoplan microscope (Leica) equipped for epifluorescence and pictures were taken with Ektachrome 800–1600 or Fuji P1600 films.

### Flow Cytometry

For three-color surface staining, thymocyte suspensions were stained with anti-CD4-PE (clone CT-CD4; Caltag), anti-CD8-FITC (clone 53.6.7), and biotinylated anti-TCRβ chain (clone H57-597) revealed with Tricolor streptadivin (Caltag).

BrdUrd was detected as described (Pénit and Vasseur, 1993) on cells previously surface stained with anti-CD44 or anti-CD4-PE and biotinylated anti-CD25 or anti-CD8 revealed by Tricolor streptavidin. Briefly, thymocytes were fixed in 1% paraformaldehyde plus 0.01% Tween 20, then treated with DNAse I (Sigma), and finally incubated with the anti-BrdUrd antibody and goat anti-mouse IgG1-FITC.

The analysis was performed with a FACScan (Becton Dickinson) and the results expressed as percentage of BrdUrd⁺ cells in each subset or as percentage of each subset in BrdUrd⁺ cells.

### ACKNOWLEDGMENTS

We wish to thank F. Alt for providing mutant mice, S. Ezine for help in bone-marrow-cell transfer, T. Ternynck for the 76-7 anti-BrdUrd antibody, and R. Hellio for confocal microscopy.

*(Received October 17, 1995)*

*(Accepted January 9, 1996)*

### REFERENCES

Bosma M.J., and Carroll A.M. (1991). The SCID mouse mutant: Definition, characterization, and potential use. Ann. Rev. Immunol. **9**: 323–350.

Godfrey D., Kennedy J., Suda T., and Zlotnik A. (1993). A developmental pathway involving four phenotypically and functionally distinct subsets of $CD3^-CD4^-CD8^-$ triple negative adult mouse thymocytes defined by CD44 and CD25 expression. J. Immunol. **150**: 4244–4252.

Hilbert D.M., Holmes K.L., Anderson A.O., and Rudikoff S. (1993). Long-term thymic reconstitution by peripheral CD4 and CD8 single-positive lymphocytes. Eur. J. Immunol. **23**: 2412–2418.

Holländer G.A., Wang B., Nichogiannopoulou A., Platenburg P.P., Van Ewijk W., Burakoff S.J., Gutierrez-Ramos J.-C., and Terhorst C. (1995). Developmental control point in induction of thymic cortex regulated by a subpopulation of prothymocytes. Nature **373**: 350–353.

Jenkinson E.J., Anderson G., and Owen J.J.T. (1992). Studies on T cell maturation on defined thymic stromal cell population in vitro. J. Exp. Med. **176**: 845–853.

Lucas B, Vasseur F., and Penit C. (1993). Normal sequence of phenotypic transitions in one cohort of 5-bromo-2'-deoxyuridine-pulse-labeled thymocytes. J. Immunol. **151**: 4574–4582.

Lucas B., Vasseur F., and Penit C. (1994). Production, selection, and maturation of thymocytes with high surface density of TcR. J. Immunol. **153**: 53–62.

Mombaerts P., Iacomini J., Johnson R.S., Herrup K., Tonegawa S., and Papaioannou V.E. (1992). RAG-1 deficient mice have no mature B and T lymphocytes. Cell **68**: 869.

Pénit C. (1986). In vivo thymocyte maturation. BUdr labeling of cycling thymocytes and phenotypic analysis of their progeny support the single lineage model. J. Immunol **137**: 2115–2121.

Pénit C. (1988). Localization and phenotype of cycling and post-cycling murine thymocyte studied by simultaneous detection of bromodeoxyuridine and surface antigens. J. Histochem. Cytochem. **36**: 473–478.

Pénit C., and Ezine S. (1989). Cell proliferation and thymocyte subset reconstitution in sublethally irradiated mice: Compared kinetics of endogenous and intrathymically transferred progenitors. Proc. Natl Acad. Sci. USA **86**: 5547–5551.

Pénit C., Lucas B., and Vasseur F. (1995). Cell expansion and growth arrest phases during the transition from precursor ($CD4^-8^-$) to immature ($CD4^+8^+$) thymocytes in normal and genetically modified mice. J. Immunol. **154**: 5103–5113.

Pénit C., and Vasseur F. (1988). Sequential events in thymocyte differentiation and thymus regeneration revealed by a combination of bromodeoxyuridine DNA labeling and antimitotic drug treatment. J. Immunol. **140**: 3315–3323.

Pénit C., and Vasseur F. (1993). Phenotype analysis of cycling and postcycling thymocytes: Evaluation of detection methods of BrdUrd and surface proteins. Cytometry **14**: 757–763.

Ritter M.A., and Boyd R.L. (1993). Development in the thymus: It takes two for a tango. Immunol. Today **14**: 462–469.

Shinkai Y., Rathbun G., Lam K.-P., Oltz E.M., Stewart V., Mendelsohn M., Charron J., Datta M., Young F., Stall A.M., and Alt F.W. (1992). Rag-2-deficient mice lack mature lymphocytes owing to inability to initiate V(D)J rearrangement. Cell **68**: 855–867.

Shores E.W., Van Ewijk W., and Singer A. (1991). Disorganisation and restoration of thymic medullary epithelial cells in T cell receptor-negative scid mice: Evidence that receptor-bearing lymphocytes influence maturation of the thymic microenvironment. Eur. J. Immunol. **21**: 1657–1661.

Surh C.D., Ernst B., and Sprent J. (1992). Growth of epithelial cells in the thymic medulla is under the control of mature cells. J. Exp. Med. **176**: 611–616.

Van Ewijk W. (1991). T cell differentiation is influenced by thymic microenvironments. Ann. Rev. Immunol. **9**: 591–615.

